# Metropolitan Hospitals and Vivisection

**Published:** 1906-03-10

**Authors:** 


### HOSPITAL ADMINISTRATION.

### CONSTRUCTION AND ECONOMICS.

### METROPOLITAN HOSPITALS AND VIVISECTION.

[We have received the subjoined letter from Mr. Stephen Coleridge, 92 Victoria Street, S.W., dated 5th inst.

It will be observed that Mr. Stephen Coleridge's letter consists of assumptions and assertions. He wrongly assumes that we cannot and do not deny that the financial transactions of many of the metropolitan hospitals, as revealed by Sir Edward Fry's Commission, constitute "gross irregularities." Mr. Coleridge rests his charge of "gross irregularities" upon the system pursued in the years preceding the Commission's appointment. The absence of justification for this charge on Mr. Stephen Coleridge's part will be apparent from the following paragraph, taken from the Report of Sir Edward Fry's Commission:—

"The great uncertainty which, in many or all cases, attends any attempt to ascertain the intentions of the original founders of the hospitals and medical schools, the impossibility of knowing what were, from time to time, the exact motives in the minds of the subsequent donors and subscribers, and the complexity of the relations which have existed between hospitals and schools, seem to render any attempt to open up the transactions in the past between the hospitals and schools very undesirable, even if it were possible."

Sir Edward Fry's Commission did not in fact report that any hospital had been guilty of any such "gross irregularity" in its financial transactions. Every reasonable man with a knowledge of the facts is satisfied with the settlement which has resulted from the findings of Sir Edward Fry's Commission. The fact that Mr. Coleridge alone expresses dissatisfaction and indulges in abuse of the managers of what he calls "the peccant hospitals" proves the settlement arrived at to be reasonable and satisfactory. Mr. Coleridge asserts that he alone is entitled to claim credit "for the preservation of these ancient charities from utterly unjustifiable desecration." If Mr. Coleridge means by this that he was the first to raise the question of the relations between the medical schools and the hospitals, and that by his action alone Sir Edward Fry's Commission was appointed, the recorded facts prove that this assumption does not rest upon any foundation which will bear the test of evidence. An Anti-vivisection Society not under Mr. Stephen Coleridge's control has already brought this fact out clearly in the press.—Ed. The Hospital.]

The letter is as follows:—

In your article which criticises my "Guide for the Charitable" you say that I describe "the financial transactions of many of the Metropolitan hospitals, as revealed by Sir Edward Fry's Commission" as "grossly irregular." So I do, and you are unable to deny those gross irregularities. I have stated that until exposed by that Commission many of these gross irregularities were successfully concealed from the public eye in the published audited accounts. This you do not, and cannot, deny. I have warned the public that as long as the same dignitaries manage these hospitals' affairs in the future as did so in the past, these irregularities are likely to continue. This you do not, and cannot, deny. You say that King Edward's Hospital Fund and the Hospital Sunday Fund have taken steps to see that the money they respectively give to hospitals shall really be spent on the sick poor and nothing else, and you complain that I have not mentioned that fact. If I had thought that the steps so far taken by these two funds had really precluded the possibility of future irregularities, similar to those I have exposed in the past, I should have said so. The peccant hospitals have, however, with almost complete unanimity proceeded to invent devices for evading the findings of Sir Edward Fry's Commission, which devices, as far as I am aware, have met with no word of condemnation from the Council of King Edward's Hospital Fund or the constituents of the Hospital Sunday Fund.

My view of the matter is that the irregularities were discovered by me, and not by the august Council and constituents of the two funds, that those august bodies would have left those irregularities to flourish had I not, to their great disgust, continually denounced them in public. Then at last the Council of the King's Fund were reluctantly forced to institute an inquiry into my charges. That although their own Commission endorsed my charges and recommended that what I had denounced should cease, the Council of King Edward's Hospital Fund expressed no condemnation of the authors of the exposed irregularities, and no reprobation of the schemes set on foot to dish the Commission and its recommendations. I very deeply regret that from the beginning of this agitation, against gross irregularities at some of the London hospitals, until now, it has been left to so humble an individual as myself to take up alone the fight for the preservation of these ancient charities from utterly unjustifiable depredations. And whenever the managers of either of the great funds have the courage publicly to condemn the authors of the scandals I have exposed, and honestly and earnestly to set themselves to the task of utterly terminating them in the future, I will very gladly and contentedly retire from the field. Until that happens, certainly I shall not consider that my task is done. When you call upon me to do this or that, or not to do this or that "as an honourable man," I take leave to tell you that I do not require the guidance of the Editor of The Hospital on matters of personal honour, and I remain, etc.

